# Neuroprotective Natural Products’ Regulatory Effects on Depression via Gut–Brain Axis Targeting Tryptophan

**DOI:** 10.3390/nu14163270

**Published:** 2022-08-10

**Authors:** Humna Liaqat, Amna Parveen, Sun Yeou Kim

**Affiliations:** 1 Department of Animal Science, Biotechnical Faculty, University of Ljubljana, Groblje 3, 1230 Domzale, Slovenia; 2 College of Pharmacy, Gachon University Medical Campus, No. 191, Hambakmoero, Yeonsu-gu, Incheon 21936, Korea

**Keywords:** L-tryptophan, 5-HT, metabolites, phytochemicals, gut–brain axis, neuroprotective, gastroprotective, signaling pathways

## Abstract

L-tryptophan (Trp) contributes to regulating bilateral communication of the gut–brain axis. It undergoes three major metabolic pathways, which lead to formation of kynurenine, serotonin (5-HT), and indole derivatives (under the control of the microbiota). Metabolites from the principal Trp pathway, kynurenic acid and quinolinic acid, exhibit neuroprotective activity, while picolinic acid exhibits antioxidant activity, and 5-HT modulates appetite, sleep cycle, and pain. Abnormality in Trp plays crucial roles in diseases, including depression, colitis, ulcer, and gut microbiota-related dysfunctions. To address these diseases, the use of natural products could be a favorable alternative because they are a rich source of compounds that can modulate the activity of Trp and combat various diseases through modulating different signaling pathways, including the gut microbiota, kynurenine pathway, and serotonin pathway. Alterations in the signaling cascade pathways via different phytochemicals may help us explore the deep relationships of the gut–brain axis to study neuroprotection. This review highlights the roles of natural products and their metabolites targeting Trp in different diseases. Additionally, the role of Trp metabolites in the regulation of neuroprotective and gastroprotective activities is discussed. This study compiles the literature on novel, potent neuroprotective agents and their action mechanisms in the gut–brain axis and proposes prospective future studies to identify more pharmaceuticals based on signaling pathways targeting Trp.

## 1. Introduction

The gut–brain axis (GBA) is a two-directional communication system between the gastrointestinal tract (GIT) and the brain. It links the emotional and cognitive centers of the brain with peripheral control and function of the GIT. Cross-communication between the gut and the brain via complex functions of neuronal, hormonal, and immune reflexes [1] is constant. This bidirectional system drives sensory signals from the gut to the central nervous system (CNS) and vice versa, thereby directing the regulation of reflex action and mood states. In turn, signals from both directions may influence motor, secretory, and immunological functions of the gut [2].

Various studies have revealed that alterations in the gut microbiota affect the GBA, which is regulated via L-tryptophan (Trp) metabolism [3,4,5]. L-tryptophan and its metabolites exhibit neuroprotective and gastroprotective activities against psychiatric disorders, such as depression, and GIT dysfunction in different diseases [6,7]. Recently, different studies have reported the regulatory roles of microbiota and their modification via dietary supplements, diet, and naturally derived agents in GBA communication [5,8]. These studies found that the gut microbiota, CNS, enteric nervous system, sympathetic and parasympathetic nervous systems, neuroendocrine system, and neuroimmune pathways are correlated. In addition, homeostasis preservation, which results from normal gut–brain communication, is disrupted in various diseases [2]. Another study revealed that changes in microbiota composition and gut dysbiosis are associated with various diseases, such as neuropsychiatric conditions, neurodevelopmental disorders, Alzheimer’s disease, Parkinson’s disease, and multiple sclerosis [9]. From this perspective, any changes in the GBA may lead to brain and gastrointestinal disorders [2], including inflammatory and irritable bowel disorders related to neuropsychiatric disorders (depression, Huntington’s disease, Parkinson’s disease, and multiple sclerosis). Accordingly, it is important to devise strategies to control different diseases associated with the GBA.

According to the World Health Organization (WHO), depression and suicide are, respectively, the primary and secondary leading causes of mental disabilities and death among individuals aged 15–29 years. Globally, more than 240 million people are affected by depression [10] and are recommended antidepressants. Although these antidepressants significantly relieve suffering, however, suicidal thoughts can be an unwanted outcome. Therefore, to combat all unwanted outcomes and to explore more potential nutritional resources, diet, and dietary supplements along with a deep understanding of the mechanisms related to this bidirectional system, the exploration of naturally occurring pharmacological agents with tryptophan-targeted therapeutic effects is necessary to develop treatment and prevention strategies for brain-related dysfunctions via the gut pathway. The bioactive compounds from natural products, such as biobased materials, bodily fluids, biomaterials, or any other natural materials, have additional advantages, such as nutritional value, easy accessibility, affordability, effectiveness, and multiple-target delivery via interlinked causative factors. Different natural products have diverse active compounds; hence, they represent a favorable alternative for targeting different interlinked factors of the GBA, with quick recovery and minimal side effects. All these factors contribute to the compliance, satisfaction, and overall health of patients.

The main aim of this review is to provide a better understanding of antidepressive natural products and their derivatives via the gut–brain axis targeting Trp. Depending on this better understanding, this review seeks to unfold various natural therapeutic agents that can interact with the causative factors of GBA-related abnormalities for neuroprotection. Additionally, the positive aspects of this study related to GBA-related abnormalities for neuroprotection, such as depression via tryptophan, are highlighted.

## 2. Materials and Methods

To achieve our goal and aims, a comprehensive literature review was studied using different electronic databases, including Web of Science, Google Scholar, and PubMed, to obtain a collection of recently published reports. We explored those reports on natural products and their derivatives or constituents that may target and involve GBA-related abnormalities for neuroprotection, such as depression via tryptophan, in recent years.

## 3. Results

### 3.1. Tryptophan Analog Structure and Biochemistry

Trp is the only amino acid (AA) derived from indole, which is a bicyclic ring formed by benzene and a pyrrole group linked to the α-carbon by a –CH2 group. The presence of the indole ring increases the hydrophobicity of the AA. Due to the high hydrophobic π electron surface area, there are various interactions between the aromatic ring and other side chains, befitting its strategic location in the protein structure [11]. The indole on the β-carbon in Trp is aromatic with a binuclear ring structure, whereas histidine and tyrosine are single-ring aromatics. The other isomeric form of Trp, designated as D-Trp, is synthesized by microbes during food processing via mRNA translation [12] and is an important biosynthetic precursor for many metabolites in microbes and their hosts. With merely 10–20% of Trp being circulated in the free form, it is the least abundant AA in the cell, and one of the rarest in the proteome; therefore, humans rely on exogenous sources of Trp (dietary intake). In contrast to animals and humans, bacteria and plants can synthesize high amounts of Trp from shikimic acid or anthranilate.

The most common sources of Trp include dairy products, oats, bananas, dried prunes, tuna fish, bread, poultry, peanuts, and chocolate. An intake of 4 mg/kg/day of Trp is recommended by the WHO [13]. The free form of Trp is also found in breast milk, playing an essential role in the infant’s postnatal development [14]. The amino acid is involved in diverse physiological processes, including cell growth, neuronal function, immunity, gut homeostasis, and host protein synthesis. Various physiologic functions are modulated by Trp, which reflects the complex actions occurring in diseases associated with adapted homeostasis [15].

### 3.2. Signaling Pathway of Tryptophan in the Gut

**Figure 1:**
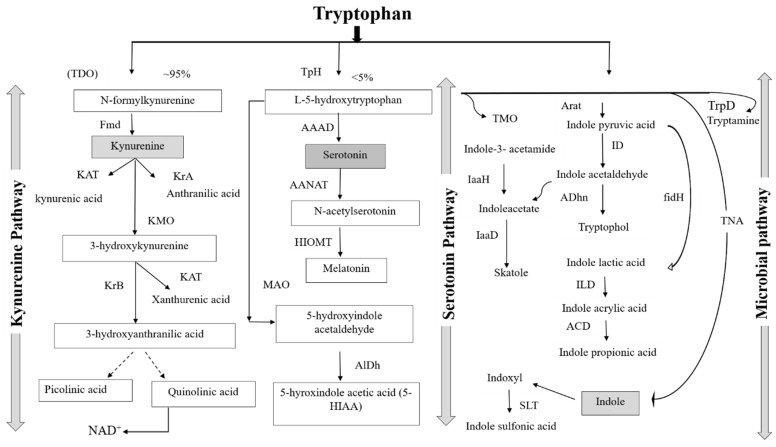
Three Integrated pathways of tryptophan metabolism. Tryptophan (Trp) is an essential amino acid obtained from dietary protein. The majority of Trp is metabolized alongside the kynurenine pathway to produce different molecules collectively referred to as kynurenines. The most widely studied fate of Trp is the downstream conversion to serotonin and melatonin. Trp availability is also altered by gut microbes generating either indole or its derivatives, tryptamine or serotonin, which can affect the gastrointestinal tract. TDO, tryptophan 2,3-dioxygenase; IDO, indoleamine 2,3-dioxygenase; Fmd, formamidase; KAT, kynurenine aminotransferase; KrA, kynureninase A; KMO, kynurenine hydroxylase (monooxygenase); KrB, kynureninase B; NAD, nicotinamide adenine dinucleotides; TpH, tryptophan hydroxylase; AAAD, aromatic amine acid decarboxylase; AANAT, arylakylamine-N-acetyltransferase; HIOMT, hydroxyindolo-O-methyltransferase; MAO, monoamine oxidase; AlDh, aldehyde dehydrogenase; TrpD, tryptophan decarboxylase; TNA, tryptophanase; SLT, sulfotransferase; TMO, tryptophan monooxygenase; IaaH, indoleacetamide hydrolase; IaaD, indoleacetate decarboxylase; Arat, aromatic amino acid aminotransferase; ID, indolepyruvate decarboxylase; ADhn, alcohol dehydrogenase; fldH, phenyllactate dehydrogenase; ILD, indolelactate dehydratase; ACD, acyl-CoA dehydrogenase.

The human GIT is inhabited by numerous commensal bacteria referred to as the gut microbiota. In recent years, Trp metabolism has emerged as a central hub for the metabolic control of neuropsychological and immunological processes. Trp plays a crucial role in conserving the balance between intestinal immune tolerance and gut microbiota maintenance [15]. The amino acid is metabolized via three different pathways in the gastrointestinal tract, namely, the direct metabolism of Trp, including ligands of the aryl hydrocarbon receptor (AhR) [16] by gut microbiota; the kynurenine pathway in both immune and epithelial cells [17]; and the serotonin pathway (5-hydroxytryptamine (5-HT)) in enterochromaffin cells [13]. All three pathways of Trp metabolism are depicted in Figure 1. In addition to endogenous Trp metabolism, resident gut microbiota contribute to the generation of specific Trp metabolites and concomitantly influence host physiology.

It has been suggested that Trp can be recycled from the intestinal epithelial cells, potentially providing a Trp reservoir. In addition, the high levels of microbiota-derived indole in the large intestine may expedite Trp formation from indole, resulting in the in situ synthesis of AhR-sensitive Trp metabolites [18].

#### 3.2.1. The Role of Tryptophan Targeting Microorganisms in the Gut

The microbiota is a complex dynamic community of microorganisms with a rich pool of genetic materials. It plays an important role in the physiological and developmental processes of humans [19]. Microbiota are present in different organs of the body, such as the GIT, skin, oral cavity, respiratory tract, and vagina. The composition of the microbiota is a significant factor in determining their positive or negative roles in human health [20]. The GIT has the largest community of microbes, with a total population of approximately 1014 cells consisting of 1000 microbial species. The gut microbiota of a typical healthy adult comprises four main phyla: Verrucomicrobia, Firmicutes, Bacteroidetes, and Actinobacteria. Age, antibiotic intake, diet, genes, infection, probiotics, prebiotics, and other chemical and physical stresses are numerous factors that affect the microbial community and their growth in the GIT and may alter the signaling pathways linked with the GBA. For example, the composition of the microbial community changes in favor of Bacteroidetes in the gut of older people compared to young people [21].

Trp metabolism by the gut microbiota is an important signaling pathway, and crosstalk between the GI epithelium and enteric flora contributes to the regulation of hormones and immune responses. This is important for the maintenance of systemic homeostasis and health. The metabolism of Trp involves a direct transfer from the intestines to the gut by intestinal microbes, partially limiting its availability. Approximately 5% of Trp is metabolized to indican, tryptamine, skatole, indole, and its derivatives. These derivatives include indole-3-aldehyde (IAld), indole-3-acid-acetic (IAA), indole-3-propionic acid (IPA), indole-3-acetaldehyde (IAAld), indole-3-ethanol (tryptophol), indole pyruvic acid, indole acetaldehyde, indole pyruvic acid, indole acetaldehyde, and indole acrylic acid (IA), which are ligands for AhR [22]. Tryptophol is a strong sleep-promoting agent that causes “sleeping sickness” due to its ability to cross the blood–brain barrier (BBB). It is produced mainly by plants and lower eukaryotes (yeast, fungi, and parasites). Indole, a major intercellular signal within the gut microbiota, is the main metabolite synthesized by gut microbes using the enzyme tryptophanase via the Ehrlich pathway, a biosynthetic pathway named after its discovery by Felix Ehrlich [23]. Indole plays a major role in the survival of microbes and controls diverse physiological processes, such as antimicrobial response, biofilm formation, motility, and a range of other functions; however, it cannot be synthesized by animal cells [24]. There is evidence that indole and its derivatives can affect both the peripheral and cerebral systems through binding to certain receptors, such as AhR [25], promoting the expression of inflammation-associated genes. Some indole derivatives, including oxindole and isatin, are characterized by neurodepressive properties, and excessive indole formation by the gut microbiota was reported to adversely affect behavior in rats [25].

Although different microbes have different catalytic enzymes, cooperation among more than two microbes is needed to produce a single metabolite from Trp [13]. Reports have shown that microbiota can directly and indirectly influence host Trp metabolism and the serotonergic system. Variations in Trp metabolism can negatively influence host microbial proliferation and microbiota diversity [26,27].

Many studies have reported that gut microbial species produce a variety of Trp metabolites through different metabolic pathways. For example, in *Lactobacillus* spp., Trp is metabolized to IAld and ILA using aromatic amino acid aminotransferase (Arat) and indole lactic acid dehydrogenase [28,29]. Similarly, *Clostridium sporogenes* convert Trp into tryptamine, ILA, and IPA [30]. Various species of *Peptostreptococcus*, including *P. russellii*, *P. anaerobius*, and *P. stomatis*, are capable of converting Trp to IA and IPA [31].

#### 3.2.2. The Role of Tryptophan Targeting the Kynurenine Pathway in the Gut

The kynurenine metabolic pathway in the gut is the most important and major catabolic route of Trp metabolism in mammals. Almost 95% of free Trp undergoes oxidative metabolism along the kynurenine pathway. Two enzymes, tryptophan 2,3-dioxygenase (TDO, highly expressed in the liver) and indoleamine 2,3-dioxygenase 1 (IDO1, expressed extrahepatically), produce several metabolites with distinct biological activities related to immune response and neurotransmission [32].

IDO is an immunoregulatory enzyme that maintains homeostasis by negatively regulating the immune system. This ferroprotoporphyrin monomeric enzyme is specific to the catabolism of Trp outside the liver [33]. In the case of inflammation or infection, a direct consequence of upregulated IDO expression is local Trp shortage surrounding T cells and an increase in kynurenine expression [7]. Generally, IDO is more nonspecific than TDO and catabolizes indole amines other than Trp. The key role of the gut microbiota in stimulating IDO1 activity has been demonstrated in germ-free and antibiotic-treated mice [34].

Following the synthesis of kynurenine from Trp, the downstream catabolic process may be divided into different pathways, leading to the formation of 3-hydroxykynurenine, anthranilic acid, or kynurenic acid (KnA). The concentration of KnA gradually increases in the IT and exhibits mucosal protective and immunoregulatory effects through its G protein-coupled receptor, GPR35, which is mostly expressed in epithelial and immune cells [35]. Two other enzymes, TDO and IDO2, metabolize Trp to kynurenine, but these enzymes are not expressed in the gut and are not discussed here. 

The catabolism of 3-hydroxyanthranilic acid results in the biosynthesis of quinolinic acid (QnA), picolinic acid, and nicotinamide. In addition to the various biological activities of Trp catabolites, activation of the kynurenine pathway contributes to the nicotinamide nucleotide pool, which is critical during starvation. Nevertheless, kynurenine metabolites are interconnected with other metabolic pathways, including the picolinic acid pathway. Modulation of the tryptophan–kynurenine pathway was proposed as an indicator of a coherent metabolic shift [36]. KnA and QnA metabolites from the kynurenine pathway are called kynurenines and are inflammatory mediators.

#### 3.2.3. The Role of the Tryptophan Targeting the Serotonin Pathway in the Gut

Serotonin (also known as 5-HT) acts as a naturally occurring non-proteinogenic amino acid, a neurotransmitter in the CNS, and a hormone in the periphery. There are two Trp genes, Tph1 and Tph2. Tph1 is expressed in enterochromaffin cells of the gut and is responsible for most of the serotonin present in the blood. Tph2 is expressed entirely in serotonergic neurons of the brainstem and is responsible for serotonin production in the brain. Brain-derived serotonin acts as a neurotransmitter, while gut-derived serotonin acts as a hormone and regulates a wide variety of processes [37]. Approximately 1–2% of ingested Trp is converted to serotonin and melatonin via the serotonin pathway. Trp is metabolized to 5-hydroxytryptophan, and further decarboxylation leads to 5-HT, which is then converted to 5-hydroxy indole acetic acid (5-HIAA). As such, depletion of Trp can decrease the biosynthesis of 5-HT, which causes emotional disturbances, depression, and cognitive impairment.

In animals, serotonin is primarily found in the GIT, blood platelets, and CNS. In humans, approximately 90–95% of the total serotonin is in the enterochromaffin cells in the GIT. 5-HT is a principal gastrointestinal signaling component that transfers signals from the gut to intrinsic or extrinsic neurons, promoting intestinal peristalsis, absorption of nutrients, vasodilatation, motility, and secretion [38].

Interruption in the central and peripheral serotonergic signaling pathways results in inflammatory bowel disease (IBD) and irritable bowel syndrome (IBS) [8]. In IBS, the imbalance of microbiota is related to alterations in the gut and brain serotonin levels [39]. A study showed that bacterial products, such as short-chain fatty acids, can upregulate serotonin biosynthesis by enterochromaffin cells [40].

Melatonin, the hormone produced by the pineal gland at night, is synthetized in several organs, including sites within the GIT. It is an end-product of the serotonin pathway, which regulates the circadian rhythm. However, melatonin can also promote IDO1 activity, which may affect the regulation of the circadian rhythms by a negative feedback loop [41]. Melatonin also affects multiple molecular pathways, including immune function, apoptosis, proliferation, angiogenesis, and oxidative stress. Lack of proper sleep is a common issue that is considered serious since it affects the autonomic nervous system, endocrine system, and immune function and is also a trigger for metabolic or mental diseases. A study of the effects of melatonin on sleep deprivation in a mouse model indicated that melatonin supplementation remediated sleep-deprivation-induced mucosal injury and altered gut microbiota composition [42].

### 3.3. Signaling Pathway of Tryptophan in the Brain

#### 3.3.1. The Role of Tryptophan Targeting Microbiota in the GBA

Currently, host–microbe interactions are more consistently reflected in the context of brain function and behavior. However, establishing a mechanistic basis for the fascinating communication along the GBA has proven to be challenging. Microbial influence on the CNS may contribute to the regulation of brain development and behavior. From this perspective, the alterations in the symbiotic crosstalk between the microbiota and the host may have significant consequences, underlying the development of both gastrointestinal and brain disorders [2]. These disorders include IBS and IBD, both of which are characterized by neuropsychiatric disorders, such as depression, Huntington’s disease, Parkinson’s disease, and multiple sclerosis. Therefore, controlling and maintaining homeostasis in the GBA is important.

The GI microbiota can regulate neurotransmitter levels either through the synthesis of neurotransmitters or by regulating the formation of its precursors, for example, *Bacillus* sp., *Escherichia* sp., and *Saccharomyces* sp. can form norepinephrine [43]. Various studies have indicated that behavior, mood, and anxiety may be influenced by the GI microbiota [44,45,46]. An in vivo study reported that germ-free (GF) mice showed different social behaviors compared with normal mice [47]. Another study indicated that individuals with depressive behaviors have a different GI microbiota composition compared to healthy subjects [48,49].

It is established that various cognitive and behavioral functions are controlled by central micro RNAs (miRNAs) [50]. Previous reports indicate that the expression of miRNAs that regulate the expression of central kynurenine pathway genes is enhanced in the hippocampi of GF mice and can be normalized by microbial colonization. These results suggest that the expression of kynurenine pathway genes in the hippocampus is regulated by gut microbiota in a miRNA-dependent manner. Evidence from the published literature indicates that gut microbiota can modulate the brain kynurenine pathway by directly regulating the activity of its key enzymes. Compared with conventional mice, GF mice show decreased IDO activity, which can be normalized after recolonization with gut microbiota [51]. Another study revealed that changes in microbiota composition led to alterations in the immune system, contributing to an abnormal immune response. In addition, gut dysbiosis is associated with many diseases, such as neuropsychiatric conditions, neurodevelopmental disorders, Alzheimer’s disease, Parkinson’s disease, and multiple sclerosis [2,9].

#### 3.3.2. The Role of Tryptophan Targeting the Kynurenine Pathway in the Brain

The kynurenine pathway, an activation pathway of the intestinal brain axis, is the proposed main route of Trp degradation due to the modulation of serotonin availability. The pathway has also been implicated in various behavioral and cognitive symptoms of neurological diseases. Trp can be degraded to kynurenine, which is then catabolized to either KnA via kynurenine aminotransferases or to 3-hydroxykynurenine (3-HK) via kynurenine 3-monooxygenase, eventually forming QnA, a well-studied metabolite from the kynurenine pathway [52]. KnA is mostly located in brain astrocytes as a neuroprotective agent and is an N-methyl-D-aspartate (NMDA) antagonist that protects against excitotoxic and apoptotic effects. However, QnA and 3-HK are mostly located in brain microglia and macrophages as neurotoxic agents [53]. QnA also shows excitotoxic effects because of its NMDA receptor agonistic effect [32]. Its neurotoxic effects are exerted through various mechanisms, including the production of reactive oxygen species, destabilization of the cellular cytoskeleton, promotion of tau phosphorylation, disruption of the BBB, and autophagy. QnA enhances the inflammatory response by stimulating the production of proinflammatory mediators in astrocytes. It is reported to potentially trigger microglia via the NMDA receptor, a pathway that has been implicated in apoptosis [54].

Nevertheless, KnA, 3-HK, and QnA all have neuroactive properties and are known to modulate dopaminergic, nicotinergic, and glutamatergic neurotransmission [53,55]. QnA and KnA play an important role in maintaining the levels of oxidative stress. The imbalance between neurotoxic and neuroprotective metabolites may be the principal driver of depression, owing to the possible effects on glutamatergic neurotransmission [56]. Both KnA and QnA are involved in anxiety and stress-related disorders by either decreasing or increasing the extracellular levels of glutamate, respectively. Decreased levels of KnA have been reported in Huntington’s disease, Parkinson’s disease, and multiple sclerosis [57].

Kynurenine can cross the BBB to participate in the CNS synthesis of neuroactive metabolites, with the majority of CNS kynurenine being derived from the periphery. Plasma kynurenine levels are reliably reflected in the CNS. Since kynurenines can reach the CNS by crossing the BBB, they are regarded as neuromodulators in diverse physiological and pathological processes of brain and GI functional disorders [58].

The influx of 5-HT and Trp in the brain can be modulated by the activation of the kynurenine pathway. Since the majority of Trp is associated with the kynurenic pathway, relatively small changes in the pathway can significantly affect the influx of Trp to the brain [59]. Additionally, kynurenines may affect the neuronal signaling involved in stress-coping mechanisms. The imbalance of metabolites is associated with multiple neurodegenerative and psychiatric diseases, including Alzheimer’s disease and depression [53].

#### 3.3.3. The Role of Tryptophan Targeting the 5-HT Pathway in the Brain

The neurotransmitter 5-HT is produced in the brain from its precursor Trp, which undergoes multiple-stage enzymatic reactions involving Trp hydroxylase (TpH) and aromatic L-amino acid decarboxylase. Melatonin is also produced in this pathway by the action of hydroxyindolo-O-methyltransferase (HIMOT), as well as the end-product 5-HIAA via monoamine oxidase activity (MAOA).

It has been reported that only 1% of dietary tryptophan is used for the biosynthesis of serotonin in the brain, and nearly 1–2% of the serotonin in the CNS contributes to the regulation of mood, sleep, pain, hunger [60], memory, and learning. Drugs that alter serotonin levels in the body are used to treat a variety of psychiatric disorders, such as depression, anxiety, migraine, nausea, memory loss, obesity, Parkinson’s disease, and schizophrenia [61]. Functional MAOA and serotonin signaling pathways are associated with depression. In depressive disorders, an increase in MAOA expression results in decreased levels of serotonin and norepinephrine, which are proposed as the principal factor [62]. Furthermore, a reduced level of serotonin in the brain is associated with increased sexuality, and TpH inhibition may be a suitable approach to addressing sexual desire disorders [63].

**Figure 2:**
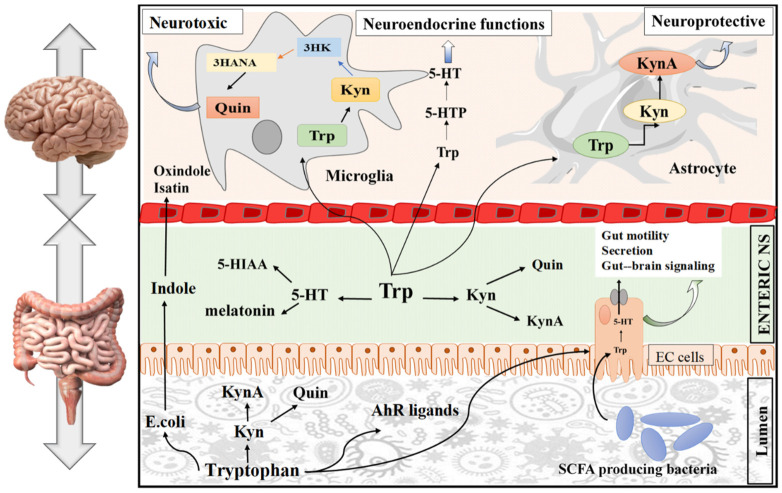
GBA targeting tryptophan: Peripheral serotonin synthesis by enterochromaffin cells is stimulated by gut microbiota. 5-HT from the gut has various direct or indirect effects, such as gut motility and gut microbiota. This affects central serotoninergic pathways by moderating Trp and tryptamine availability. Gut microbiota affect the kynurenine pathway, which plays a critical role in inflammatory mechanisms and neuroendocrine functions. Dietary Trp can also be directly converted by the gut microbiota into AhR ligands and can help to perform many functions. Trp; tryptophan; Kyn, kynurenine; KynA, kynurenic acid; quinolinic acid; 3HANA, 3-hydroxyanthranilic acid; 3-HK, 3-hydroxykynurenine; 5-HTP, 5-hydroxytryptophan, 5-HT, 5-hydroxytryptamine; 5-HIAA, 5-hydroxyindole acetic acid; AhR, aryl hydrocarbon receptor; EC, enterochromaffin cells.

A balance is needed between the bacterial utilization of Trp and the Trp requirement for serotonin production in the gut and CNS [64]. Serotonin is vital for this axis; it acts as a neurotransmitter in the CNS and in the enteric nervous system, which is present in the gut wall. Serotonin is produced by endocrine cells that act as paracrine hormones in the gut [65] and links both ends of the brain–gut axis. Serotonin can use intestinal chromaffin cells as chemoreceptors to transmit perceived chemical information to the nervous system [66], swiftly transmitting intestinal signals to the brain [67]. Thus, local changes in the GIT are affected by serotonin concentration, which thereby affects central neurotransmission [64]. An overview of the Trp function in the GBA is compiled and presented in Figure 2. This reveals that Trp is an initiator that causes changes in the gut, either directly or indirectly, via different signaling pathways. In addition to changes in the gut, contributions to abnormal functions of the brain, including neuropsychiatric conditions, neurodevelopmental disorders, Alzheimer’s disease, Parkinson’s disease, and multiple sclerosis, are observed. Therefore, in exploring the detailed mechanism in the gut–brain axis, more effective targeted therapies can be developed to overcome brain-related diseases. In addition to limiting the availability of Trp for central serotonin synthesis, activation of the kynurenine pathway also plays a vital role in the modulation of brain functions by producing downstream neurotoxic/neuroprotective metabolites [68].

### 3.4. The Role of Natural Products and Their Metabolites in the GBA Targeting Tryptophan

**Figure 3:**
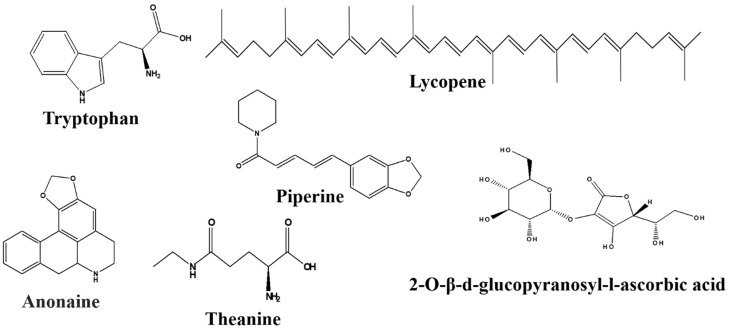
Structures of bioactive metabolites from natural products: Tryptophan—human breast milk, *Moringa oleifera*, and *Nelumbo nucifera*; theanine—*Camellia sinensis*; anonaine—*Annona muricata*; piperine—*Piper nigrum*; lycopene—*Citrullus lanatus*; 2-O-β-d-glucopyranosyl-l-ascorbic acid—*Lycium barbarum*.

**Figure 4:**
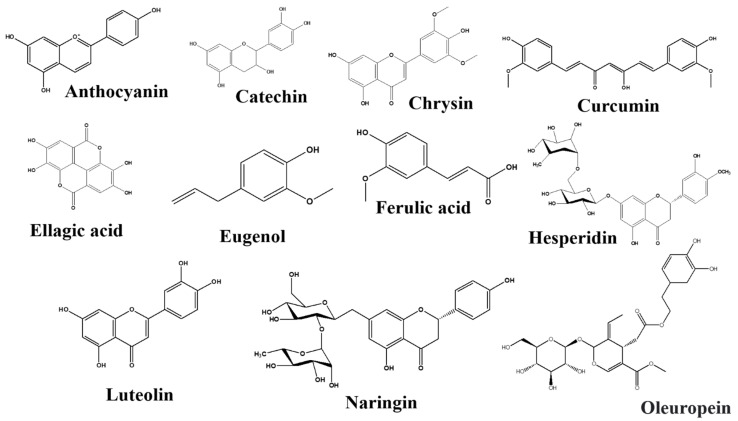
Structures of bioactive metabolites from natural products: Anthocyanin—*Rubus fruticosus*; Catechin—*Rhizophora mucronata*; Chrysin—*Matricaria chamomilla*; Curcumin—*Curcuma longa*; Ellagic acid—*Punica granatum*; Eugenol—*Syzygium aromaticum*; Ferulic acid—*Ferula foetida*; Hesperidin—*Citrus limon*; Oleuropein—*Olea europaea*.

**Figure 5:**
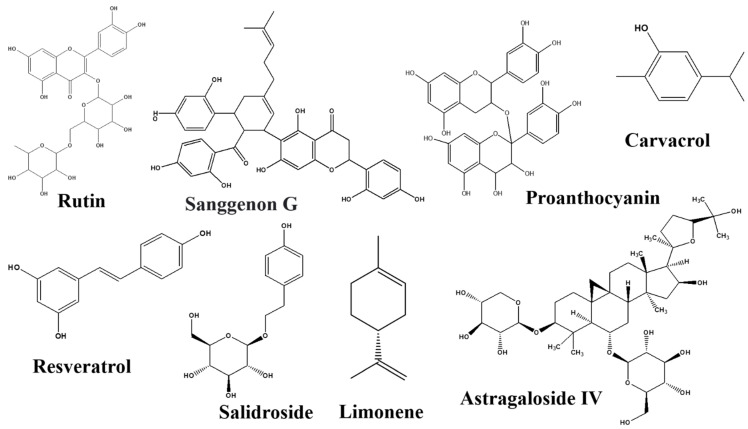
Structures of bioactive metabolites from natural products: Proanthocyanin—*Cinnamomum zeylanicum*; Rutin—*Fagopyrum esculentum*; Sanggenon G—*Morus alba*; Salidroside—*Rhodiola rosea*; Resveratrol—*Polygonum cuspidatum*; Astragaloside IV—*Astragalus membranaceus*; Carvacrol—*Origanum vulgare*; Limonene—*Citrus sinensis*.

**Figure 6:**
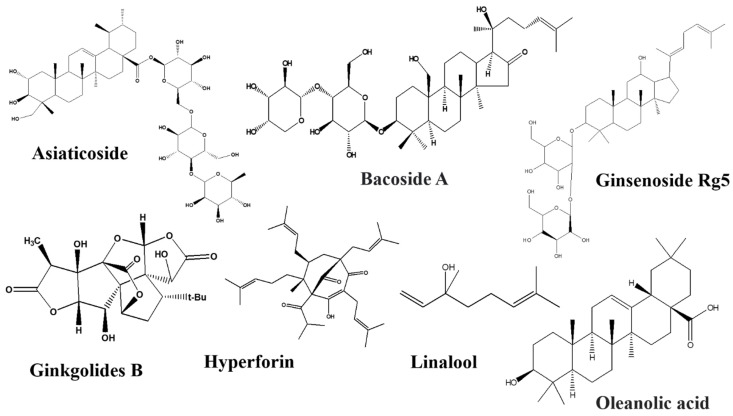
Structures of bioactive metabolites from natural products: Asiaticoside—*Centella asiatica*; Bacoside—*Bacopa monnieri*; Ginkgolides B—*Ginkgo biloba*; Linalool—*Lavandula angustifolia*; Ginsenoside Rg5—*Panax ginseng*; Oleanolic acid—*Pimenta pseudocaryophyllus*; Hyperforin—*Hypericum perforatum*.

There are numerous natural products along with their active components in the GBA that target Trp. All the natural products with their bioactive compounds, family, class, target signaling pathway, action, and effect on the GBA are summarized in the Table 1. Furthermore, the chemical structure of metabolic compounds of natural products are presented in Figure 3, Figure 4, Figure 5 and Figure 6.

#### 3.4.1. Natural Products

##### Human Breast Milk

Human breast milk (HBM) is a complex fluid, universally known as the optimal source of nutrients for infants. It contains essential nutrients and a diverse microbial population [211]. Although Trp is the primary constituent of HBM, melatonin [178,179] is also supplied to the infant via breast milk. This plays a significant role in improving the sleep cycle and reducing infantile colic [180].

A study revealed that HBM naturally contains selective serotonin reuptake inhibitors, which are widely used as antidepressants [212]. Recent studies have demonstrated that the combination of ingested HBM in conjunction with a probiotic is the best protective therapy for necrotizing enterocolitis (excessive inflammation in the intestine; NEC). ILA, a metabolite of Trp, interacts with the transcription factor AhR and prevents the transcription of the inflammatory cytokine IL-8. ILA produced by *B. infantis* and *Bacteroides fragilis* interacts with HBM, effectively treating NEC in premature infants [213,214].

##### *Moringa oleifera* 

*Moringa oleifera* (MO) is a softwood vegetable plant with reported antiulcer, antifertility, antidiabetic, and antidepressant activities [184]. Many parts of the tree are edible, and the flowers and leaves are consumed in salads, soups, sauces, tea, and as cooked vegetables. It is a natural source of Trp, which is the main agent protecting the GBA [215]. In most studies, ethanol extract of the plant was found to be the most effective [185,186]. A study revealed that MO had a protective effect against ulcers by increasing the enterochromaffin cell count and 5-HT levels via activating serotonin receptors on gastric tissues [187]. The combined administration of ethanolic MO (EMO) extract with low doses of fluoxetine or other selective serotonin reuptake inhibitors (SSRIs) showed potential as an alternative therapy in the treatment of anxiety or depression [188]. MO also acts as a neuroprotective agent; previous reports indicated that MO leaves showed a significant anxiolytic effect. However, the exact mechanism of action needs to be determined [186]. According to previous studies, EMO extract possesses an antidepressant effect and can be used for the future treatment of neurodegenerative disorders, including depression and Alzheimer’s disease [184,185].

##### *Nelumbo nucifera* 

*Nelumbo nucifera* belongs to the mono-generic family Nymphaeaceae and is commonly known as sacred lotus and water lily. All parts of N. nucifera have various medicinal uses [189]. Nelumbo nucifera contains natural Trp, which is effective in the gut–brain pathway [190]. A recent study demonstrated that *N. nucifera* possesses an antidepressant effect, which is closely linked with serotonergic mechanisms [191]. However, the exact mechanism remains to be studied. Another study claimed that N. nucifera may act as a direct 5-HT receptor agonist, enhancing the serotonergic activity of neurons by either inhibiting serotonin reuptake or activating serotonin metabolism [192]. Further research is needed to determine the mechanism for the neuroprotective and antidepressant effects of *N. nucifera*.

##### *Mimosa pudica* 

*Mimosa pudica* is commonly known as a sensitive plant (touch-me-not) [216] and has received attention from many researchers worldwide because of its pharmacological activities. These include hepatoprotective, antioxidant, antitoxin, antidiabetic, anti-inflammatory, and wound-healing activities. The medicinal plant contains various tannins, alkaloids, flavonoids, and glycosides [197]. A study reported that the anxiolytic effect of M. pudica may be due to an antagonistic effect on the 5-HT receptor. Although *M. pudica* possesses an antidepressant effect, along with memory enhancement through multiple mechanisms, the exact mechanism needs to be studied [198]. *Mimosa pudica* has a therapeutic effect in the management of Parkinson’s disease by suppressing α-synuclein and dopaminergic neurodegeneration. The constituent quercetin may be responsible for this activity because of its antioxidative mechanism [217]. *Mimosa pudica* can also regulate neuroactive ligand–receptor interaction, as well as serotonin and dopamine synapses, by modulating multiple proteins in Parkinson’s disease [199].

##### *Poria cocos* 

*Poria cocos* (PC) is an edible medicinal mushroom that is used as a complementary therapy to treat depression and anxiety owing to its anti-inflammatory properties. It significantly mitigated depression-like behaviors in chronic, unpredictable mild-stress rats, restoring brain-derived neurotrophic factor (BDNF) levels and neural growth in the hippocampus. This potential antidepressant effect is mediated by the gut microbiota and cecal contents metabolism. PC can increase biodiversity and markedly regulate the relative abundance of healthy microbes. The gut microbiota is strongly linked with cecal metabolism, especially energy metabolism, inflammation, and immunity [200].

Both PC and *Cordyceps militaris* exhibit anxiolytic and antidepressive effects by decreasing inflammation and modulating neurotransmitters. They significantly decrease glutamate and the metabolism of serotonin and dopamine in the prefrontal cortex. However, they improve serum IL-1β levels and reduce p38 mitogen-activated protein kinases (p38 MAPK) and nuclear factor kappa B (NF-κB) protein expression in the prefrontal cortex [201].

##### *Salvia officinalis* 

*Salvia officinalis* (SO), commonly known as sage, came from the Latin word meaning “to heal”. It contains rosmarinic acid and caffeic acid, which possess anxiolytic and antidepressant activities [218]. However, further studies are needed to understand how rosmarinic acid and the active compounds of SO target the Trp signaling pathway. Additionally, a study in 2018 revealed that SO improved the gut microbiota in diabetic animal models [202].

*S. officinalis* is widely used as an alternative to treat depression, digestive and circulation problems, memory issues, different inflammations, and asthma [203]. A study demonstrated that the modulation of neuroreceptors and serotonin transporters was the mode of action for *S. officinalis* extract (SOE), making it an effective alternative for remediating mental impairment and thermoregulation during menopause [204]. *Salvia officinalis* could be used as a food additive to protect against neurotoxicity to ameliorate behavioral and oxidative stress [219]. A recent study revealed that SO could be used against memory deficits in rats in which neurotoxicity was induced. SOE improves memory and behavioral activities by elevating BDNF and CREB (Ca2+/cAMP response element-binding protein) levels [220].

##### *Tagetes lucida* 

*Tagetes lucida* is an aromatic and traditional medicinal plant that is widely used in Mexico for anxiety treatment and has been recorded as an antidepressant medicinal plant in other regions of Mexico. The anxiolytic and sedative-like properties of *T. lucida* may result from the effect of its coumarinic constituents on serotonergic neurotransmission [205], manifesting an antidepressant effect by the serotonergic system, with no adverse effects [206]. The modulation of the release and reuptake of serotonin and this antidepressant effect may also be due to quercetin, a compound in *T. lucida* [207].

##### *Theobroma cacao* 

Cocoa beans (*Theobroma cacao*) have been known as “the food of the gods,” and belong to the Sterculiaceae family. Theobromine (3,7-dimethylxanthine) is the most active and abundant compound present in cocoa beans [181]. It is a rich source of Trp content with potential anti-inflammatory and antioxidant activities [182]. Chocolates are usually obtained from this plant and are widely consumed in many countries, including Switzerland and Great Britain. It interacts with neurotransmitters, such as 5-HT, which contribute to mood regulation, reward, and appetite. It is reported that chocolate reduces the risk of various diseases and boosts the immune system by altering the gut microbiota [183]. However, an exact signaling pathway needs to be identified, along with the bioactive agents present in cocoa beans.

##### Tualang Honey

Since ancient times, honey has been used to treat different diseases. Tualang honey has been extensively studied for its application in modern medicine. It is rich in polyphenols with antioxidant, antiproliferative, and wound-healing properties [221]. It enhances antidepressive effects in stressed rats by boosting BDNF concentration and restoring the hypothalamic–pituitary–adrenal axis [208]. It is reported that tualang honey protects against memory decline caused by stress or aging by changing hippocampal morphology. A reduction in oxidative stress and upregulation of BDNF concentration in the brain was also proposed as a potential mechanism of action [209]. Another benefit included the observed improvement in immediate and spatial memory in postmenopausal women and middle-aged people who consumed tualang honey, respectively [210].

Honey has been shown to improve the gut microbiome dysbiosis owing to its prebiotic alteration of the microbiota growth and antimicrobial effects [222]. Although honey is involved in the improvement of cognitive functions [223], exact mechanisms and longitudinal research are needed to understand the role of honey as a brain tonic and to identify its active constituents.

#### 3.4.2. Amino Acids

##### Theanine

Theanine (γ-glutamylethylamide) is an AA mainly derived from tea and is primarily found in green tea plants (*Camellia sinensis*). It influences many neurotransmitters in the brain, including acetylcholine, dopamine, and serotonin [174]. Theanine intake is involved in the development of hippocampal function after weaning [175] and was reported to have antistress effects [176]. Long-term administration of theanine increases BDNF levels, supporting a neuroprotective effect. An assessment of chronic, unpredictable mild-stress (CUMS) rats found that both 5-HT and dopamine levels increased after the administration of theanine, revealing an antidepressant effect [177]. The main signaling pathway ERK/CREB/BDNF was observed to modulate the GBA.

#### 3.4.3. Alkaloids

##### Anonaine and Asimilobine

Both anonaine and asimilobine are isolated from *Annona muricata* and have sedative properties. This property is observed owing to the influence of the alkaloids on the CNS via the serotonin receptors [69]. Both are the active constituents of the genus Annona (Annonaceae), which comprises approximately 82 species. Several species of Annona are used in traditional Mexican medicine for their antianxiety, anticonvulsant, and tranquilizing properties. For example, *A. purpurea*, *A. cherimolia*, *A. muricata*, *A. vepretorum*, and *A. coriacea* possess sedative, anxiolytic, and antidepressant properties. *Annona cherimolia* was revealed to have an antidepressant effect by increasing serotonin and dopamine turnover (monoaminergic turnover) [224]. The ethanol leaf extract of *A. muricata* (soursop) was also considered to be a promising neurobiological therapeutic after sedative and antidepressant-like effects were observed in Sprague Dawley rats [70]. The essential oil of *A. vepretorum* exhibited anxiolytic, sedative, and antidepressant effects. A previous study confirmed that the antidepressant effect of *A. vepretorum* was related to the serotonergic pathway and the anxiolytic effects on the GABAergic system [71]. *Annona coriacea* (Mart.), popularly known as araticum, also showed anxiolytic and antidepressant effects by regulating monoaminergic and GABAergic neurotransmitters [72].

##### Piperine

Piperine is a major alkaloid constituent of *Piper nigrum* (black pepper) and has been widely and traditionally used as a flavoring agent; it is also used as a table condiment. Multiple studies have revealed the neuroprotective effects of this alkaloid against various neurodegenerative disorders [157,158,159]. Additionally, it exhibits an antidepressant effect in CUMS-treated mice and ameliorates CUMS-induced downregulation of BDNF expression in the hippocampus and frontal cortex [225]. Co-administration of trans-resveratrol and piperine exerts an antidepressant-like effect via the inhibition of MAOA activities, which regulate the levels of neurotransmitters such as 5-HT in the brain, thereby further activating the 5-HT and BDNF signaling pathways [160]. Furthermore, piperine remediates cognitive impairment in mouse models through antioxidant and anti-inflammatory effects, thereby restoring neurotransmission [158]. Nevertheless, identification of potential mechanisms underlying the neuroprotective effects of dietary piperine is needed.

#### 3.4.4. Carotenoids

##### Lycopene

Lycopene is an aliphatic hydrocarbon carotenoid that is found in various fruits and vegetables, including watermelon (*Citrullus lanatus*) and tomatoes (*Solanum lycopersicum*). Lycopene possesses strong antioxidant activities compared to carotenoids and exerts neuroprotective effects [142]. It improves posttraumatic stress disorder (PTSD)-like behavior in mice by rebalancing the neuroinflammatory response and oxidative stress in the brain. The restoration of BDNF expression may be a potential mechanism underlying the anti-PTSD effects of lycopene [142].

Another study reported the antioxidant and neuroprotective effects of lycopene against acrylamide-induced neurotoxicity in rats [142]. Lycopene administration elevated the levels of antioxidants (reduced glutathione reductase and glutathione peroxidase) and neurotransmitters (serotonin and dopamine) and reduced the expression of oxidative stress biomarkers (malondialdehyde, nitric oxide, and protein carbonyl), thereby reversing the effect of acrylamide [143].

##### 2-O-β-d-glucopyranosyl-l-ascorbic Acid

2-O-β-d-glucopyranosyl-l-ascorbic acid is the active compound of *Lycium barbarum* (LB), which regulates the gut microbiota [193]. LB is used as a traditional medicine in some Asian countries. It has neuroprotective, antiaging, and antioxidant properties and contains various polyphenols, which are responsible for its neuroprotective effects [226]. LB has been implicated in the reduction in anxiety and depression-like behaviors, which may be mediated by the enhancement of synaptic plasticity [194]. The antidepressant effect of LB is also attributed to its strong antioxidative properties, which lead to the reduction in apoptosis in striatum neurons [195]. A human trial study reported the novel role of LB in increasing serotonin levels and proposed it as a complementary treatment for depression [227]. Furthermore, LB is involved in the regulation of the gut microbiota. A recent study demonstrated that LB maintains organism health by regulating the gut microbiota [193,196].

#### 3.4.5. Flavonoids and Phenolics

##### Anthocyanins

Anthocyanins are ubiquitous flavonoids present in different fruits and vegetables, including Russian Boc Thorn (*Lycium ruthenicum*), cauliflower (*Brassica oleracea*), and blackberry (*Rubus fruticosus*). Anthocyanins are known for their antioxidant, anti-inflammatory, antiaging, and antibacterial properties [73]. A study conducted to explore the effects of anthocyanins on brain aging revealed an increase in the levels of serotonin, norepinephrine, and dopamine, suggesting that anthocyanins may be used to maintain cognitive and memory function in aging mice [74]. A recent study proposed that anthocyanins ameliorate depression-like behavior in CUMS rats by increasing the levels of monoamine neurotransmitters, upregulating BDNF expression, and inhibiting MAOA, which promotes neurogenesis [75]. Furthermore, anthocyanins from *L. ruthenicum* exhibit dynamic and multiple regulatory effects on the intestinal microbiota and are crucial for maintaining intestinal health [76].

Additionally, anthocyanins have psychobiotic properties, as demonstrated in a study wherein dietary manipulation of the gut microbiota with anthocyanins ameliorated neurologic complications. Moreover, blackberry anthocyanin-rich extract (BE) can regulate the composition of the gut microbiota. Furthermore, modifications in the gut microbiota were reported to be partially linked to the anti-neuroinflammatory properties of BE. Reportedly, BE modifies the host Trp metabolism via the kynurenine pathway and increases the synthesis of the neuroprotective metabolite KnA [77].

##### Catechins

Catechins comprise epigallocatechin-3-gallate, epigallocatechin, epicatechin-3-gallate, and epicatechin, which are major polyphenolic compounds in green tea. Catechins mitigate anxiety and provide mood-related benefits by modulating BDNF levels and the pro-BDNF and monoaminergic signaling pathways [90]. Bifidobacterium spp. exhibit beneficial psychobiotic effects, including attenuation of anxiety responses, daily reported stress levels, and cortisol output, in humans [91]. Furthermore, catechins suppress MAOA in mouse brain mitochondria. 

Both catechins and epicatechins have therapeutic effects against neurodegenerative disorders and block QnA-induced lipid peroxidation while recovering the QnA-induced altered oxidized glutathione balance [92]. These compounds reportedly showed antioxidative activities in an excitotoxic model, which supports the active role of glutathione as an antioxidant, and could potentially be used in the treatment of neurodegenerative diseases [93]. For example, catechin-rich *Rhizophora mucronate* significantly reduces the neurotoxicity induced due to a beta-amyloid peptide (Aβ)-associated oxidative stress and could be a potent drug for the treatment of Alzheimer’s disease [92]. Recent studies indicate that epigallocatechin gallate causes microbial degradation, enters the brain parenchyma via the blood–brain barrier, and suppresses brain dysfunction [94].

##### Chrysin

Chrysin (5,7-dihydroxyflavone or 5,7-dihydroxy-2-phenyl-4H-chromen-4-one) is a flavonoid found in honey, propolis, blue passionflower (*Passiflora caerulea*), and *Matricaria chamomilla*. It has attracted substantial attention for its medicinal properties against several disorders, including neurodegenerative disorders, owing to its multiple mechanisms of action [95]. Chrysin improves 3-nitropropinoic acid-induced neurotoxicity by targeting MAOA and 5-HT levels and improves histological alterations associated with Huntington’s disease symptoms in rats [96].

Chrysin exhibits a protective effect against cisplatin (cis-diamminedichloroplatinum (II)) CDDP-induced jejunum toxicity by mitigating oxidative stress and apoptotic tissue damage [97]. Similarly, it is proposed that chrysin alleviates isoniazid-induced brain oxidative damage, inflammation, and apoptosis via its antioxidant properties [98]. Although chrysin has antioxidant and antiapoptotic functions in the brain and gut, the exact signaling pathway and their interconnection mechanisms are unknown, indicating a need for further research.

##### Curcumin

Curcumin is an essential curcuminoid present in turmeric (*Curcuma longa*) and is more commonly known in Asia as the golden spice. It is traditionally used for relieving mental stress, hypochondriac distension, and mania and is also a part of oriental medicine. It elicits antioxidant, anti-inflammatory, and anticancer effects. Curcumin downregulates the production of kynurenine from Trp and increases the synthesis of 5-HT [99]. As a known antidepressant, it regulates and elevates serotonin levels in the hippocampus region [100]. A study demonstrated that the administration of curcumin (20 and 40 mg/kg) increased 5-HT levels in mice. The antidepressant effect of curcumin could be increased if used in combination with other antidepressants, such as bupropion and desipramine [228]. Another study demonstrated that curcumin normalized the QnA/Trp ratio via the inhibition of stress-induced overexpression of indoleamine 2,3-dioxygenase in rats [101]. Additionally, the novel curcumin derivative J147 exhibits antidepressant-like effects by increasing 5-HT and BDNF levels in the hippocampus of mice [102].

The antidepressant action of curcumin may be related to an increase in hippocampal BDNF expression, which directly correlates with the pathophysiology of depression. A recent study revealed that curcumin could act as a potential protective agent against sodium salicylate and gentamicin-induced neurotoxicity and neurobehavioral aberrations by regulating apoptotic pathways owing to its antioxidant properties. Sodium salicylate and gentamicin result in GABA depletion, oxidative damage, and apoptotic alterations in the brain, and curcumin can significantly reverse these adverse effects [229].

##### Ellagic Acid

Ellagic acid (EA) (2,3,7,8-tetrahydroxy-chromeno [5,4,3-cde]chromene-5,10-dione) is a natural phenolic compound present in various fruits and nuts, such as blueberries, blackberries, raspberries, strawberries, longan seeds, and walnuts [103], and is the active ingredient of pomegranate (*Punica granatum*). EA is known for its antioxidant, anti-inflammatory, and anticancer effects. Additionally, it has neuroprotective effects [104], as it protects brain tissues against ACR-induced neurotoxicity by mitigating inflammation and oxidative stress [230]. EA improves CUMS-induced depression-like behavior by regulating BDNF and 5-HT levels and suppressing the secretion of proinflammatory serum cytokines [105]. Furthermore, it targets the Trp–kynurenine pathway, regulates the Trp microbial and host metabolism in mouse and human models, and alters neural activity to improve memory function [106].

##### Eugenol

Eugenol (EU; 4-allyl-2-methoxyphenol) is an active compound of cloves (*Syzygium aromaticum*) and is widely distributed in different plants such as basil, turmeric, pepper, ginger, oregano, cinnamon, and thyme [107]. EU exhibits neuroprotective activity against excitotoxic and oxidative injury by modulating both NMDA receptors and superoxide radicals [108]. This is attributed to its anti-inflammatory and antioxidant activities. Furthermore, EU protects against stress-induced IBS-like gastrointestinal dysfunction by modulating the brain’s monoaminergic pathways, including serotonin, norepinephrine, and dopamine pathways [109]. It also exhibits a neurorestorative effect in streptozotocin-diabetic rats by reducing oxidative stress and increasing glutathione levels [231]. A recent study demonstrated that EU exhibits therapeutic effects against neurotoxicity owing to its antioxidant and antiapoptotic activities [110].

##### Ferulic Acid

Ferulic acid (4-hydroxy-3-methoxycinnamic acid; FA) is obtained from *Ferula foetida*, a spice used as a digestive aid and in foods such as pickles and condiments. It is a phenolic acid present in the leaves and seeds of different plants, including pineapples, rice, oats, oranges, wheat, and artichokes [111]. It has a wide range of medicinal properties, including neuroprotective, hepatoprotective, anti-inflammatory, antidiabetic, anticarcinogenic, antiapoptotic, antiaging, antiatherogenic, hypotensive, and vasodilatory effects [112].

FA exhibits therapeutic effects against neurotoxicity induced by acrylamide in rats [113]. A study provided evidence indicating that FA elevates the levels of 5-HT and norepinephrine in the hippocampi and frontal cortices of mice. These variations may be ascribed to the inhibition of MAOA activity [114]. A study reported that its anti-inflammatory mechanism is involved in the observed antidepressant-like effects of FA in stressed mice [115]. The production of bioactive derivatives of FA depends on the gut microbiota. FA is reported to improve depressive-like behavior via upregulation of BDNF, synapsin I, and postsynaptic protein PSD95 levels in the hippocampus and prefrontal cortex [232]. However, the exact mechanism of FA and its effects on 5-HT and MAOA are unknown and require further study.

##### Hesperidin

Hesperidin (3,5,7-trihydroxy-4–methoxy-flavanone-7-rhamnoglucoside) is a flavanone glycoside and a dominant flavonoid in citrus species such as *Citrus limon* [123]. A study demonstrated that hesperidin can reduce neuroinflammation and increase BDNF synthesis in the hippocampus [124]. Furthermore, it is known to ameliorate mild stress-induced depression by suppressing inflammation and microglial activation in the prefrontal cortices of rats [233]. A study showed that hesperidin improves neural function, reduces oxidative stress and inflammation, and upregulates BDNF levels [125], thereby confirming its protective effects against neurotoxicity and neuroinflammation. A recent study reported that hesperidin regulates GI motility, increases the metabolism of the gut microbiota, and induces the biosynthesis of hesperetin-derived metabolites [234]. Accordingly, it may be beneficial to explore hesperidin as a GI motility-regulating drug [126].

##### Luteolin

Luteolin (3′,4′,5,7-tetrahydroxyflavone) is one of the most common flavonoids present in edible plants (*Eclipta prostrata*, *Arachis hypogaea*, and chamomile tea), fruits (apple skins, oranges, grapefruit, and lemons), and vegetables (broccoli, parsley, pepper, thyme, carrot, and celery) [138,139]. The antidepressant-like effect of luteolin is attributed to the downregulation of the plasma membrane monoamine transporter and the increase in BDNF expression. It was reported to directly or indirectly inhibit serotonin reuptake, resulting in an increase in MAO neurotransmitter levels in the synaptic cleft [140]. Luteolin reduces *Caenorhabditis elegans* fat storage by promoting the central 5-HT signaling pathway. Treatment with luteolin elevates the expression of tph-1 (Trp hydroxylase) and increases the mRNA levels of mod-1 and ser-6, which are the serotonin-related receptors that play vital roles in serotonin-mediated fat reduction. Furthermore, luteolin elevates serotonin synthesis in neurons to promote lipolysis and fatty acid β-oxidation in *C. elegans* [141].

##### Naringin

Naringin is a well-studied plant secondary metabolite that naturally exists in grapefruit (*Citrus paradise*) and other citrus fruits and has numerous biological benefits. Clinical evidence has confirmed the therapeutic potential of naringin in the prevention of hypertension, diabetes, and neurodegeneration via antioxidant and anti-inflammatory activities [145]. Naringin exhibits neuroprotective effects against QnA-induced neurotoxicity through the modulation of apoptotic markers, oxidative stress, mitochondrial complex, and neuroinflammatory activities. QnA-induced neurotoxicity alters the levels of apoptotic markers (Bax, Bcl-2, PPAR-γ mRNA, and caspase-3), oxidative stress (superoxide dismutase, nitric oxide, glutathione, and malondialdehyde), and neuroinflammatory markers (TNF-α, ILs, and NF-kB mRNA), and this effect is significantly (*p* < 0.05) mitigated by naringin [146]. Naringin could be a potential therapeutic alternative for the treatment of Huntington’s disease-like symptoms.

##### Oleuropein

Oleuropein is a phenolic compound found in the Oleaceae family (*Olea europaea*). Oleuropein showed neuroprotective effects in a Parkinson’s cell model and prevented neural death and reduced oxidative stress in a neuronal dopaminergic cellular model [151]. Recent studies have indicated that it modulates the gut microbiota and affects the mucosal immune system. Furthermore, it increases the levels of anti-inflammatory cytokines, such as TGF-β and IL-10, and T-regulatory cells, which helps in inflammation suppression and enhances immune tolerance to bacteria and other harmless dietary antigens. It also promotes the formation of intestinal IgA, which protects against pathogenic bacteria and enhances the homeostasis of the gut microbiota. This affects cognitive health by promoting the growth of *Lactobacilli* sp. and *Bifidobacterium* sp., which produce gamma-aminobutyric acid, the main inhibitory neurotransmitter. An increase in the population of *Lactobacilli* sp. leads to the production of aryl hydrocarbon receptor ligands from Trp, thereby affecting the mucosal immunity status [152]. The exact pathway underlying oleuropein’s action is still unknown, and further studies are needed to understand its role in the GBA signaling pathway.

##### Proanthocyanidins

Proanthocyanidins are oligomeric and polymeric flavan-3-ols found in various plants, including cinnamon (*Cinnamomum zeylanicum*). It possesses antioxidant, antinociceptive, and neuroprotective properties. Administration of proanthocyanidins led to a marked increase in 5-HT levels in three brain regions: the frontal cortex, hippocampus, and hypothalamus in the human brain [161]. A recent study claimed that proanthocyanidins have a therapeutic role in adolescent depression, wherein it improves depression-like behavior and increases the number of hippocampal neurons [162].

##### Rutin

Rutin (3,30,40,5,7-pentahydroxyflavone-3-rhamnoglucoside), the most ubiquitous flavonoid, is the primary glycoside form of quercetin and is present in different foods and beverages, including buckwheat (*Fagopyrum esculentum*), apples, onions, red wine, and tea [166]. Rutin has potential therapeutic applications in neurodegenerative disorders. This flavonoid has a neuroprotective effect and reduces cisplatin-induced neurotoxicity owing to its antioxidant properties [200,201]. A previous study revealed that rutin reduces colistin-induced neurotoxicity in male rats. The administration of colistin increased BDNF levels, oxidative impairment, malondialdehyde content, and the levels of apoptotic and inflammatory factors (Bcl-2 associated X protein (Bax), cysteine aspartate-specific protease-3 (caspase-3), tumor necrosis factor-α (TNF-α), B-cell lymphoma-2 (Bcl-2), NF-κB, and neuronal nitric oxide synthase (nNOS)). This indicates that rutin restores brain function by mitigating colistin-induced inflammation, oxidative stress, apoptosis, and histopathological changes [167]. Reportedly, rutin also exerted an antidepressant-like effect in a mouse model for maternal separation stress via NMDA receptors [235].

##### Sanggenon G

Sanggenon G is a novel, natural, non-peptidic, and active constituent of *Morus alba*, which has been substantially used in traditional medicine. M. alba roots (MAR) possess significant antistress properties induced by chronic foot stock [171]. MAR has potential therapeutic effects on diabetes-associated depression. A study revealed that MAR can improve diabetes-induced depression by increasing BDNF levels. Sanggenon G in MAR possesses antidepressant-like effects, which are mediated by the serotonergic system [172]. MAR extracts may also act as potent novel neuroprotective and memory-enhancing agents for menopausal women [173].

##### Salidroside

Salidroside is a tyrosol glycoside isolated from *Rhodiola rosea* and is responsible for its antistress activity. It regulates the GBA by adjusting the microbiota and modulating inflammation in the CNS and peripheral circulation. Salidroside potentially reduces hippocampus-dependent memory impairment by reducing Aβ1-42 deposition, microglial activation, and expression of proinflammatory factors, such as TNF-α, IL-6, and IL-1β, in the brain. In addition, salidroside improves gut barrier integrity by modifying the gut microbiota. In peripheral circulation, it reduces the levels of proinflammatory cytokines, particularly Il-12, IL-17A, IL-6, and IL-1α [168].

*Rhodiola rosea* species are commonly known as roseroots, golden roots, or orpin rose. *R. rosea* is a well-known physical and mental booster [169] and is considered a dietary supplement owing to its several health benefits. It improves 5-HT levels in the hippocampi of depressive rats [170].

##### Resveratrol

Resveratrol (3,5,40 -trihydroxystilbene) is a phytoalexin and polyphenol predominantly present in Japanese knotweed (*Polygonum cuspidatum*), the skin of red wine, red grapes, and some nuts. Recent studies have examined the potential use of resveratrol in improving sleep quality, reducing fatigue, and subduing anxiety and depression [163]. Resveratrol inhibits the expression of sodium-dependent serotonin transporter (SERT) and increases 5-HT levels [164]. A study on the potency of resveratrol reported that it reduces anti-IBS-like effects, including hypersensitivity, intestinal motility abnormality, anxiety, and depression, by regulating the serotoninergic signaling pathway in the GBA [165]. Another study confirmed that resveratrol inhibits 5-HT reuptake and increases serotonergic function [236]. Resveratrol has been reported to be a promising alternative as a neuroprotective therapeutic agent for the treatment of Parkinson’s disease. It reduces oxidative damage via activation of the SIRT1/Akt1 signaling pathway [237]. A more recent study claimed that resveratrol mitigates neurotoxicity and reduces SIRT1 activity by regulating BDNF signaling [238].

#### 3.4.6. Terpenoids

##### Astragaloside IV

Astragaloside IV (AS-IV) is a lanolin alcohol-derived tetracyclic triterpene saponin extracted from *Astragalus membranaceus*. This plant is a member of the Leguminosae family, and its dried root is used in traditional Chinese medicine to strengthen the host defense system. Moreover, it has neuroprotective, hepatoprotective, anti-inflammatory, and antidepressant effects. *Astragalus membranaceus* polysaccharides are known to reduce neuroinflammation, which improves the behavior of metabolically stressed transgenic mice [239].

AS-IV is a potential drug for treating neurodegenerative diseases. AS-IV prevents amyloid β protein fragment 1–42 oligomer-induced memory impairment and neuronal apoptosis by promoting expression of the PPARγ/BDNF signaling pathway [240]. Co-administration of AS-IV and *A. spinosus* saponins reportedly relieved bisphenol-A-induced neuropsychiatric symptoms in a rat model of schizophrenia. These compounds mitigated memory impairment and restored the expression of neurotransmitters, including serotonin, dopamine, and MAO [241]. AS-IV reduces neuroinflammation and oxidative stress and improves oxaliplatin-induced neurotoxicity. This terpenoid decreases TNF-α, IL-6, and IL-1β levels, thereby inhibiting inflammation and reducing MDA. The increase in SOD, CAT, and GSH-Px activities blocks oxidative stress [242] to reduce neuroinflammation.

##### Asiaticoside

*Centella asiatica* (CA) is a member of the Apiaceae (Umbelliferae) family, and asiaticoside is one of the principal active compounds. It is a psychoactive medicinal plant that has been used for decades to improve symptoms of anxiety and to stimulate a profound state of mental calmness [78]. CA also suppresses the activity of QnA. According to a study in 2015, CA prevented lipid peroxidation and thiol oxidation in male adults’ brains induced by QnA (excitotoxic) [79].

CA improves cognitive performance by promoting BDNF expression in the rat prefrontal cortex [80] and has a reported therapeutic effect on gut microbiota. A recent study demonstrated the promising application of CA in the clinical treatment of ulcerative colitis. The ethanol extract of CA improved colitis induced by dextran sulfate sodium and restored gut microbiota homeostasis and the mucosal barrier. CA displayed an anti-inflammatory effect by suppressing inflammatory cell infiltration and reducing myeloperoxidase activity in the colon. Furthermore, CA restores intestinal motility by promoting c-Kit expression in the colon and increasing 5-HT levels in the brain [81].

##### Bacosides

Bacosides are the bioactive constituents of *Bacopa monnieri* that protect the brain against oxidative damage and cognitive deterioration. Bacoside A can interact with neurotransmitters, directly or indirectly, to improve memory and learning abilities [82]. B. monnieri is an herb extensively used in Ayurveda and has a therapeutic effect in treating depression. Previous reports indicate that the extracts of *B. monnieri* (BME) ameliorated chronic, unpredictable stress in depressive rats by regulating BDNF levels in the hippocampus [83]. BME improved recognition by increasing cell proliferation and neuroblast differentiation in the dentate gyrus, which led to elevated BDNF levels [84]. Another study suggested that BME could be an effective alternative for treating prenatal stress-induced behavioral impairment in Wistar rat offspring. Exposure to BME affects the serotonergic system via altering the expression of synaptic proteins and serotonin receptors and through the interconversion of pro and mature BDNF. This treatment protects against neural damage and changes in pro and mature BDNF, which may be linked to the observed anxiolytic behavior in offspring [85].

##### Carvacrol

Carvacrol (5-isopropyl-2-methylphenol) is a monoterpenic phenol present in the essential oil *Origanum vulgare*. This aromatic phytochemical is known for its neuroprotective, gastroprotective, cardioprotective, hepatoprotective, anticancer, anti-inflammatory, antidiabetic, antiarthritic, antioxidant, antiallergic, analgesic, and sedative properties [86]. A study suggested that carvacrol is a brain-active molecule, and administration of this phenol significantly increases 5-HT contents in the prefrontal cortex and hippocampus [87]. Carvacrol shows inhibitory effects against memory degeneration in neurodegenerative diseases. It protects against brain tissue inflammation and oxidative stress in lipopolysaccharide-challenged rats [88]. A recent study revealed that carvacrol can mitigate memory impairment caused by neuroinflammation. This memory and cognitive enhancement are mediated by the regulation of BDNF due to the anti-inflammatory effects of carvacrol [89].

##### Ginkgolides

*G. biloba* (GB) is a popular herbal medicine, and studies have indicated its hepatoprotective, anti-inflammatory, antioxidant, and cardioprotective effects [116]. Ginkgolides, including ginkgolides A, B, C, J, K, L, and M, are unique terpenoid components of GB and have neuroprotective effects against oxidative stress, inflammation, apoptosis, and cognitive impairment [117]. Ginkgolide B is reported to reduce myocardial infarction-induced depression-like behaviors [118], but the exact mechanisms of ginkgolide B and all other forms (A, C, J, K, L, and M) remain largely unknown.

Various preclinical and clinical studies have shown a positive effect of GB in improving depression and cognitive abilities and reducing anxiety under pathological conditions [243,244]. *G. biloba* extract (GBE) can ameliorate the cognitive function of rats with vascular dementia. GBE increases serotonin, dopamine, and acetylcholine levels and inhibits the activity of acetylcholinesterase (ACHE) [245]. In combination with depressive drugs, GBE plays a synergistic role, and the onset efficacy time is faster than that of single antidepressants [246]. It exerts antidepression effects via modulating hippocampal BDNF expression [247] and the gut microbiome through water-soluble polysaccharides [119]. GBE also exerted an antioxidative effect in the hippocampus of ovariectomized rats by restoring the serotonin (5-HT1A and 5-HT1B) and leptin receptor levels [248].

##### Linalool

Linalool is one of the main constituents of lavender and coriander oil. This natural therapeutic agent exhibits protective effects against neurotoxicity induced by Aβ1-42 [134]. Lavender oil is obtained from *Lavandula angustifolia* (commonly known as English lavender or Ustukhuddoos). It has long been used in Iranian traditional medicine for treating nervous disorders, such as depression and epilepsy. This aromatic herb has potent sedative, mood-stabilizing, antidepressant, and anxiolytic properties [135]. A study revealed that lavender oil acts as an antidepressant by reducing anxiety and sleep disturbances in depressive patients [136]. Linalool, limonene, and α-pinene are the primary bioactive components responsible for the activity of lavender oil, which inhibit Trp breakdown in a dose-dependent manner. The main signaling therapeutic pathways include the GTP-CHI and IDO pathways; another study revealed that the anxiolytic-like effect of lavender essential oil is mediated by 5-HT transmission [137].

##### Ginsenosides Rb1 and Rg5

Ginsenosides Rb1 and Rg5 are the most common bioactive compounds in *Panax ginseng* (PG). Panax ginseng (PG) is known as the king of Korean and Chinese herbal medicines and has been widely used for treating CNS disorders. PG may also be used as a natural alternative to antidepressant drugs to treat menopausal depression, as it activates serotonergic neurons under stress [249]. The long-term use of PG is considered safe, with minimal adverse side effects [250]. The ginsenosides Rb1 and Rg5 exhibit antidepressant activities and regulate serotonergic, dopaminergic, and BDNF levels [120,121,122]. Another study revealed that 5-HT enhances the sleep-improving effects of Rb1 and Rg5, both of which upregulated 5-HT1A expression in a rodent model. The main signaling pathways involved are the GABAergic and serotoninergic signaling pathways [251]. 

##### Limonene

Limonene is a cyclic terpene found in citrus fruits. It can inhibit stimulant-induced behavioral changes by regulating dopamine levels and 5-HT receptor function [131]. It is an active compound of *Citrus sinensis*, a sweet orange belonging to the Rutaceae family, and is a rich source of flavanones. A study reported that functional compounds in orange juice could reduce IBD through various mechanisms. Orange juice also aids in the prevention and treatment of colon inflammation and the prevention of oxidative damage [132]. A randomized controlled study was conducted to evaluate the effect of orange juice on the GBA. This study reported that flavonoid-rich orange juice alters the gut microbiome and improves symptoms of depression in young adults. It is also known to increase BDNF levels in young adults with depression symptoms. However, the precise signaling pathway needs to be identified, along with the bioactive agents present in *C. sinensis*. Flavonoids are known to reduce inflammatory reactions and provide neuroprotection [133]. During alcoholic fermentation of pomegranate, malt, and grapes, melatonin synthesis occurs, which improves their health benefits; similarly, orange juice can be subjected to alcoholic fermentation, and it can be determined whether the fermented juice affects the GBA effects [133].

##### Oleanolic Acid

Oleanolic acid (OA) and (E)-methyl isoeugenol (MIE) are the major active compounds isolated from *Pimenta pseudocaryophyllus* and exhibit antidepressant effects via the inhibition of MAOA [147]. *P. pseudocaryophyllus*, most commonly used as a diuretic and flavoring agent, is native to Brazil and has promising pharmacological activity [148]. A previous study revealed that the dichloromethane fraction of *P. pseudocaryophyllus* shows antidepressant activity via targeting Trp [252]. Another study demonstrated that the administration of OA and MIE from *P. pseudocaryophyllus* increased BDNF levels, and the anxiolytic and antidepressant activities depended on monoamine biosynthesis and serotonergic pathways [149,150].

#### 3.4.7. Fatty Acids

##### Omega-3 Fatty Acid

Fish oil (FO) is a rich source of omega-3 essential fatty acids that cannot be synthesized by mammals. It shows a therapeutic effect against different diseases, particularly mood-related disorders, and improves memory. The administration of FO reportedly reduced the negative behavior induced by lipopolysaccharide in aged mice via modulation of the kynurenine and serotonergic pathways [153]. FO also exhibited antidepressant-like effects in postpartum depression patients via modulating the serotonergic system [253]. Omega-3-fatty acid-rich FO also minimizes anxiolytic and antidepressant behaviors and improves memory function via regulating BDNF concentrations [154]. A recent study revealed that FO exhibits an antidepressant-like effect and prevents lipopolysaccharide-induced depressive behavior by reducing IDO expression and increasing serotonin levels [155]. Mitigation of postpartum depression by reducing neuroinflammation and modifying the hypothalamic–pituitary–adrenal axis via the serotonergic pathway was also observed [156].

#### 3.4.8. Phloroglucinol Derivative

##### Hyperforin

*H. perforatum* is a perennial herbaceous plant that is widely known as St. John’s wort. It has long been used as a traditional herbal medicine owing to its analgesic and healing effects [254]. It exhibits an antidepressant effect via activating the serotonin transporter on neurons. The underlying mechanism for the activation of serotonin involves *H. perforatum*-induced reduction in corticosterone and TNF-α levels. However, no effect on the kynurenine/Trp ratio was observed [127]. Furthermore, it was revealed that *H. perforatum* exerted potent antidepressant effects in postmenopausal women by acting as an MAOA inhibitor and upregulating 5-HT2 receptors [128]. Hypericin and hyperforin are the active compounds of *Hypericum perforatum* that exhibit antidepressant effects. Hyperforin can efficiently inhibit the reuptake of L-glutamate, serotonin, GABA, dopamine, and noradrenaline [129,130].

## 4. Discussion

The GBA is a two-sided communication network between the GIT and CNS. Tryptophan, an essential amino acid, plays a vital role in the normal growth and health of both humans and animals by exerting modulatory effects at multiple levels. It is the only serotonin precursor, and once consumed, it is distributed throughout the body via the circulatory system. Disruption in its composition in the gut has been reported to be associated with several pathophysiological conditions, including diabetes, obesity, colorectal cancer, inflammatory bowel diseases, and neurological disorders via different signaling pathways [255,256]. Some of the neurological disorders that can be targeted via the gut–brain axis are depression and stress. Depression can result in suicidal thoughts in various age groups. Extensive studies are being conducted to obtain more details regarding the mechanisms involved in the gut–brain axis as well as to identify naturally available resources, for example, dietary supplements and edible and medicinal plants, as alternative treatments. Success from these investigations could help in resolving the related issues and improving public health. Identifying various novel natural products that interact with and affect the GBA could further have an added value. Therefore, first of all, to achieve a better understanding of the role of Trp, the development of reliable models to interpret the complex interactions between the gut microbiota and disruptions in dietary metabolism, which ultimately affect brain functionality, mood, and behaviors, is necessary. Accordingly, this review aimed to provide a comprehensive overview of the complicated communication between the GBA to aid better understanding of pathways interlinked with each other, either directly or indirectly. Moreover, while researching pathways, we came across natural products that not only affect the interlinked GBA factors but also play therapeutic roles in GBA-related diseases. Therefore, this review not only highlights the positive aspects of previous research but also opens the door to future research for researchers and scientists in order to give a clear picture of controlling depression via the GBA using various natural products and their derivatives, as mentioned below.

**Figure 7:**
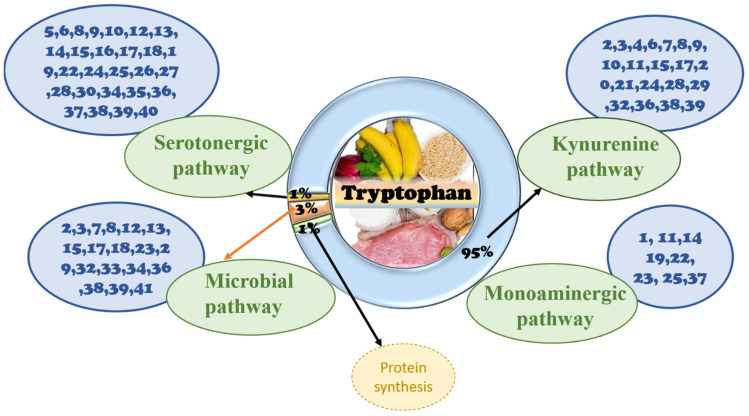
Metabolism of Trp by natural products and constituents via different signaling pathways: Trp is metabolized via the kynurenine pathway (95%), serotonin pathway (1%), and microbial pathway (3%). 1: anonaine, 2: anthocyanin, 3: asiaticoside, 4: astragaloside IV, 5: bacoside A, 6: carvacrol, 7: catechin, 8: chrysin, 9: curcumin, 10: ellagic acid, 11: eugenol, 12: ferulic acid, 13: ginkgolides B, 14: ginsenoside Rg5, 15: hesperidin, 16: hyperforin, 17: limonene, 18: linalool, 19: luteolin, 20: lycopene, 21: naringin, 22: oleanolic acid, 23: oleuropein, 24: omega-3 fatty acids, 25: piperine, 26: proanthocyanidins, 27: resveratrol, 28: rutin, 29: salidroside, 30: sanggenon G, 31: theanine, 32–35: tryptophan, 36: 2-O-β-d-glucopyranosyl-l-ascorbic acid, 37: *Mimosa pudica*, 38: *Poria cocos*, 39: *Salvia officinalis*, 40: *Tagetes lucida*, 41: Tualang honey.

In the major kynurenine pathway, KnA is neuroprotective, while QnA is excitotoxic [52]. Inflammatory processes and oxidative stress trigger a disturbance between the neurotoxic and neuroprotective branches of the kynurenic pathway. This leads to complications related to the brain, such as depression and anxiety [64]. Serotonin transfers intestinal signals to the brain, along with playing multiple roles in different parts of the body, and its modulation could provide ample therapeutic opportunities for multiple diseases, including depression, anxiety, migraine, nausea, phobia, memory loss, obesity, Parkinson’s disease, and schizophrenia [61]. Melatonin may affect the autonomic nervous system, endocrine system, immune function, and sleeping cycle, which can act as a trigger for metabolic or mental diseases, implicating it as a beneficial metabolite for the brain [42]. The overall summary of natural products and their constituents and the various pathways for Trp metabolism is shown in Figure 7.

Tryptophan is considered an initiator of the gut microbiota, the kynurenine pathway, and the serotonin pathway; therefore, studying these pathways targeting tryptophan can help in developing strategies for targeted delivery therapy. According to the WHO report, depression will be the largest global burden disease in 2030. More than 50% of people suffer from depression and anxiety [201]. The most prescribed treatments for depression include serotonin reuptake inhibitors, antidepressants, serotonin and norepinephrine inhibitors, and monoamine oxidase inhibitors. In addition, as a natural sedative, Trp has a profound therapeutic effect in combating anxiety and depression caused by various conditions such as IBD [132], postmenopause [128], postpartum [253], and posttraumatic stress [142]. Although these therapies are quite effective, they prolong the suffering of patients, which may result in suicidal thoughts. Therefore, discovering natural products to identify serotonin inhibitors or tryptophan-rich sources may aid in finding alternative treatments with minimal side effects. In addition, identifying such natural products targeting depression may prove beneficial in developed countries, including Japan and South Korea, where the depression-related suicide rate is quite high [257,258]. Human breast milk, *M. oleifera*, and *N. nucifera* are rich sources of tryptophan. The literature indicates that both limonene and human breast milk target the melatonin signaling pathways, ultimately improving brain health via inhibiting the mTOR through the MT1 receptor [257,258]. Therefore, adding M. oleifera and N. nucifera to the daily diet may help to alleviate depression [178,179,215].

Considering the adverse effects of modern medicine, alternatives such as food and natural supplements can be an attractive option as a source of Trp and for the treatment of depression. For example, theanine [177], curcumin [102], ellagic acid [105], ginsenoside Rb1 [251], hesperidin [233], anthocyanins [75], piperine [160], and omega-3 fatty acids [156] from green tea, turmeric, pomegranate, ginseng, lemon, blackberry, black pepper, and fish oil may all be used. Orange juice and its alcoholic fermented orange beverages [144,259], *G. biloba* leaves [116], FA [111], carvacrol [87], and chrysin [95] are used as traditional therapeutic medicines in different regions of the world to overcome stress and depression.

Several studies have been conducted to determine the potential effects of natural products on depression and neuroprotection; however, the constituents of these resources remain largely unknown. Future studies are necessary to identify the constituents of tualang honey [209,223], *P. cocos* [201], *S. officinalis* [202], *M. pudica* [199], *T. lucida* [207], and *C. sinensis* [132]. A previous study revealed that carvacrol possesses neuromodulatory properties; however, there is a need to study its potential clinical efficacy as well as its toxicity prior to any recommendation for its use [87]. Recent studies on the microbial pathway in the gut revealed that individuals with high indole production by the gut microbiota are more likely to experience anxiety and mood disorders. Thus, food or natural substances promoting indole production should be avoided in people who are already suffering from anxiety and mood disorders.

Most of the studies discussed in this review were focused on Trp and its metabolites, targeting the gut–brain signaling pathway. The exact mechanism of Trp in the gut is still unknown in most phytochemicals (chrysin, luteolin, lycopene, naringin, piperine, proanthocyanidins, quercetin, resveratrol, rutin, theanine, and curcumin) and medicinal plants (*H. perforatum*, *E. prostrata*, *M. alba*, *N. nucifera*, *Annona* spp., *O. vulgare*, *B. monnieri*, and *A. membranaceus*). Some natural products show activity against the GBA targeting intestinal immunity; however, the exact signaling pathway needs to be studied to determine whether it can affect the Trp signaling pathway.

Although the bioactive compounds of various medicinal plants remain unknown and need to be further explored, some studies have reported the expected constituents that may be responsible for the neuroprotective effects to reduce depression or gastroprotective activities of some natural products. One study reported that *M. pudica* shows a therapeutic effect in the management of Parkinson’s disease, and quercetin may be responsible for this activity owing to its antioxidative mechanism [217]. The antidepressant effect of *T. lucida* may be due to the presence of quercetin [207]. Similarly, *S. officinalis* possesses rosmarinic acid and caffeic acid, which have anxiolytic and antidepressant activities [218]. However, the exact Trp signaling pathway needs to be studied deeply to target rosmarinic acid and its active compounds.

**Figure 8:**
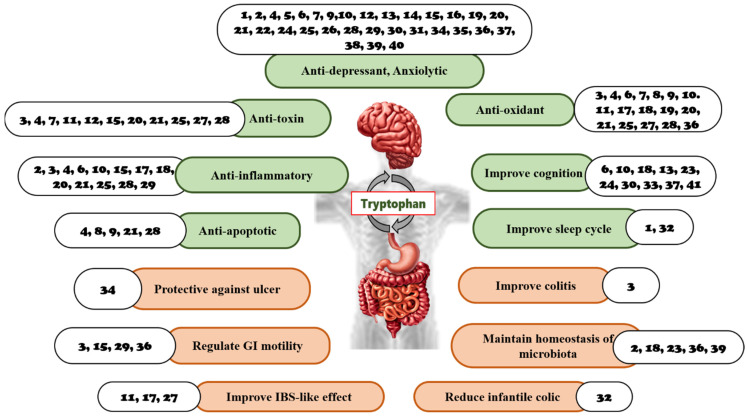
Natural products with neuroprotective and gastroprotective effects on the regulation of the GBA targeting tryptophan and its metabolites through different pathways: Specific natural products and the derived compounds exhibit specific functions in the bidirectional communication of the gut and brain. 1: anonaine, 2: anthocyanin, 3: asiaticoside, 4: astragaloside IV, 5: bacoside A, 6: carvacrol, 7: catechin, 8: chrysin, 9: curcumin, 10: ellagic acid, 11: eugenol, 12: ferulic acid, 13: ginkgolides B, 14: ginsenoside Rg5, 15: hesperidin, 16: hyperforin, 17: limonene, 18: linalool, 19: luteolin, 20: lycopene, 21: naringin, 22: oleanolic acid, 23: oleuropein, 24: omega-3 fatty acids, 25: piperine, 26: proanthocyanidins, 27: resveratrol, 28: rutin, 29: salidroside, 30: sanggenon G, 31: theanine, 32–35: tryptophan, 36: 2-O-β-d-glucopyranosyl-l-ascorbic acid, 37: *Mimosa pudica*, 38: *Poria cocos*, 39: *Salvia officinalis*, 40: *Tagetes lucida*, 41: Tualang honey.

The nutritionally essential amino acid Trp contributes to the regulation of numerous physiological mechanisms, including serving as a precursor for the neurotransmitter serotonin. Melatonin, which is an important metabolite of the serotonin pathway, also needs to be studied because of its prominent association with the sleeping cycle, oxidative stress, and apoptosis. Many rich natural products targeting the serotonin and kynurenine signaling pathways, along with their metabolites, have been studied for over a decade, but the indole pathway and its metabolites remain to be studied in the context of natural resources. Figure 8 presents an overall summary of various natural therapeutic agents and their effects on the brain and gut.

Based on the information presented in this review, we conclude that natural products and their metabolites exert gastroprotective and neuroprotective effects via three pathways. Overall, ellagic acid and rutin were found to be the most potent compounds after natural tryptophan. These compounds target different factors and are particularly involved in the prevention/treatment of psychological diseases. Additionally, depression can be alleviated by controlling tryptophan levels in the GBA. Importantly, the serotonin pathway should be given more consideration to unfold more potential natural product treatment alternatives. There is an urgent need for further studies on the signaling pathways to develop affordable and accessible therapeutic alternatives for the prevention and treatment of gut- and brain-related disorders, particularly those related to depression.

## Figures and Tables

**Table 1:** Natural products and the derived potential compounds that exhibit gastroprotective and neuroprotective effects through regulating the GBA targeting tryptophan.

Compound	Origin	Targeted Pathway	Family	Class	Pharmacological Target	Pharmacological Action	References
Anonaine	*Annona muricata*	Monoaminergic	*Annonaceae*	Alkaloid	5-HT	Antidepressant, sedative, and anxiolytic	[69,70,71,72]
Anthocyanins	*Rubus fruticosus*	Microbial, kynurenine, MAO	*Rosaceae*	Flavonoid	BNDF, 5-HT, gut	Anti-neuroinflammatory, antidepressant, prevents brain aging, and regulates the gut microbiota	[73,74,75,76,77]
Asiaticoside	*Centella asiatica*	Kynurenine, microbial	*Apiaceae*	Terpenoid	BDNF, QnA, inflammatory markers, microbiota homeostasis, and mucosal barrier	Prevents neurotoxicity, lipid peroxidation, neuroinflammation; improves colitis, GI motility, and homeostasis	[78,79,80,81]
Astragaloside IV	*Astragalus membranaceus*	BDNF, kynurenine	*Fabaceae*	Polyphenol	5-HT, dopamine, and MAO levels; oxidative, apoptotic, and inflammatory parameters	Neuroprotection against toxicity, inflammation, oxidative stress, apoptosis, and depression	[78,79,80,81]
Bacoside A	*Bacopa monnieri*	Serotonergic system	*Plantaginaceae*	Terpenoid	BDNF, 5-HT receptors, and synaptic proteins	Antidepressant and anti-anxiolytic	[82,83,84,85]
Carvacrol	*Origanum vulgare*	Serotoninergic, kynurenine	*Lamiaceae*	Phenol	5-HT and BDNF	Gastroprotective and provides neuroprotection against memory degeneration, inflammation, oxidative stress, and depression	[86,87,88,89]
Catechins	*Rhizophora mucronata*	Microbial, kynurenine	*Rhizophoraceae*	Flavonoid	QnA, BDNF, microbes, and MAO	Neuroprotection against anxiety, oxidative stress, and neurotoxicity	[90,91,92,93,94]
Chrysin	*Matricaria chamomilla*	Kynurenine, serotoninergic, and microbial	*Asteraceae*	Flavonoid	MAO and 5-HT	Gut and neuroprotection against oxidative stress, apoptosis, and inflammation	[95,96,97,98]
Curcumin	*Curcuma longa*	Kynurenic, serotoninergic	*Zingiberaceae*	Phenol	5-HT, kynurenine, QnA, BDNF	Neuroprotection against oxidative stress, apoptosis, and depression	[99,100,101,102]
Ellagic acid	*Punica granatum*	Kynurenine, serotoninergic	*Lythraceae*	Phenol	5-HT and BDNF	Neuroprotection against inflammation, oxidative stress, and depression; improves memory	[103,104,105,106]
Eugenol	*Syzygium aromaticum*	Monoaminergic, kynurenine	*Myrtaceae*	Phenol		Neuroprotection against toxicity, oxidative stress, and IBS-induced stress	[107,108,109,110]
Ferulic acid	*Ferula foetida*	Serotoninergic, microbial	*Poaceae*	Phenol	5-HT, MAOA, and BDNF	Elevates 5-HT levels and has antidepressant and anti-neurotoxic effects	[111,112,113,114,115]
Ginkgolides B	*Ginkgo biloba*	Serotonergic, microbial	*Ginkgoaceae*	Terpenoid	BNDF and 5-HT	Reduces depression and anxiety and improves cognitive abilities	[116,117,118,119]
Ginsenoside Rg5	*Panax ginseng*	Serotonergic, dopaminergic, GABAergic system	*Araliaceae*	Terpenoid	BNDF and 5-HT	Antidepressant	[120,121,122]
Hesperidin	*Citrus limon*	Serotonergic, kynurenine, microbial	*Rutaceae*	Flavonoid	BDNF	Regulates GI motility and provides neuroprotection against toxicity, inflammation, and depression	[123,124,125,126]
Hyperforin	*Hypericum perforatum*	MAOA, serotonergic system	*Hypericaceae*	Terpenoid	5-HT, MAOA, and the kynurenine/Trp ratio	Antidepressant	[127,128,129,130]
Limonene	*Citrus sinensis*	Kynurenine, serotonergic, microbial	*Rutaceae*	Terpene	Melatonin, BDNF, and gut microbiome	Neuroprotection against inflammation and oxidative stress (IBD)	[131,132,133]
Linalool	*Lavandula angustifolia*	Serotonergic system, microbial	*Lamiaceae*	Terpenoid	5-HT, gut microbiota, inflammatory markers, and mucosal immunity	Neuroprotection against inflammation, oxidative stress, and neural death; improves gut microbiota homeostasis and cognitive health	[134,135,136,137]
Luteolin	*Eclipta prostrata*	Monoaminergic, serotonergic	*Asteraceae*	Flavonoid	MAO neurotransmitters, 5-HT-related receptors, BDNF, 5-HT, and monoamine transporter	Antidepressant-like effect, inhibits serotonin reuptake, and promotes lipolysis and fatty acid β-oxidation	[138,139,140,141]
Lycopene	*Citrullus lanatus*	BDNF, kynurenine	*Cucurbitaceae*	Carotenoid	BDNF, serotonin, dopamine, inflammatory, and oxidative markers	Neuroprotection against inflammation, oxidative stress, toxicity, and stress	[142,143,144]
Naringin	*Citrus paradisi*	Kynurenine	*Rutaceae*	Flavonoid	Neuroinflammatory, apoptotic, and oxidative markers	Neuroprotection against inflammation, oxidative stress, toxicity, and stress	[145,146]
Oleanolic acid	*Pimenta pseudocaryophyllus*	Monoaminergic, serotonergic system	*Myrtaceae*	Terpenoid	5-HT and MAOA	Anxiolytic, antidepressant	[147,148,149,150]
Oleuropein	*Olea europaea*	Dopaminergic, microbial system	*Oleaceae*	Phenol	Inflammatory markers, gut microbiota, and 5-HT	Neuroprotection against inflammation, improvement of the gut microbiota homeostasis and cognitive health	[151,152]
Omega-3 fatty acids	Fish oil	Kynurenine or serotonergic		Fatty acid	BDNF, serotonin, and IDO	Antidepressant and anti-anxiolytic; improves memory	[153,154,155,156]
Piperine	*Piper nigrum*	Monoaminergic, serotonergic, BDNF	*Piperaceae*	Alkaloid	5-HT and MAOA	Neuroprotection against inflammation, oxidative stress, toxicity, and stress	[157,158,159,160]
Proanthocyanidins	*Cinnamomum zeylanicum*	Serotonergic	*Lauraceae*	Polyphenol	5-HT	Antidepressant	[161,162]
Resveratrol	*Polygonum cuspidatum*	Serotoninergic	*Polygonaceae*	Polyphenol	5-HT, SERT, and BDNF	Enhances 5-HT levels, inhibits 5-HT reuptake, provides neuroprotection against toxicity, oxidative damage, and IBS-like effect	[163,164,165]
Rutin	*Fagopyrum esculentum*	Kynurenine, serotonergic	*Polygonaceae*	Flavonoid	NMDA, BDNF, QnA, oxidative, apoptotic, and inflammatory parameters	Neuroprotection against inflammation, oxidative stress, apoptosis, toxicity, and depression	[166,167]
Salidroside	*Rhodiola rosea*	Microbial, kynurenine	*Crassulaceae*	Glycoside	Inflammatory markers, gut microbiota, and 5-HT	Antidepressant, regulates gut–brain axis by modulation of gut microbiota and inflammation	[168,169,170]
Sanggenon G	*Morus alba*	Serotonergic	*Moraceae*	Flavonoid	BNDF	Antidepressant, antistress agent, memory-enhancing agent	[171,172,173]
Theanine	*Camellia sinensis*	BDNF	*Theaceae*	Amino acid	5-HT, BDNF, and dopamine	Antidepressant	[174,175,176,177]
Tryptophan	Human breast milk	Kynurenine, microbial		Amino acid	AhR	Improves the sleep cycle and reduces infantile colic	[178,179,180]
Tryptophan	*Theobroma cacao*	Microbial	*Sterculiaceae*	Amino acid	5-HT and gut microbiota	Neuroprotective and improves cognition	[181,182,183]
Tryptophan	*Moringa oleifera*	Microbial, serotonergic	*Moringaceae*	Amino acid	SSRI, 5-HT, and EC cell count	Anxiolytic, antidepressant, and protects against ulcers	[184,185,186,187,188]
Tryptophan	*Nelumbo nucifera*	Serotonergic system	*Nymphaeaceae*	Amino acid	5-HT	Antidepressant, 5-HT reuptake inhibitor, and 5-HT metabolism activator	[189,190,191,192]
2-*O*-β-D-Glucopyranosyl-l-ascorbic acid	*Lycium barbarum*	Serotonergic, microbial, kynurenine	*Solanaceae*	Vitamin	Proinflammatory cytokines, 5-HT, and antioxidative markers	Reduces depression and anxiety and stabilizes the gut microbiota	[193,194,195,196]
	*Mimosa pudica*	Serotonergic, dopaminergic	*Fabaceae*		5-HT receptor	Antidepressant, memory enhancer, and regulates neuroactive ligand–receptor interaction	[197,198,199]
	*Poria cocos*	Kynurenine, serotonergic, microbial	*Polyporaceae*		BDNF, gut microbiome, and serotonin	Antidepressant, anxiolytic, and mediates the gut microbiota	[200,201]
	*Salvia officinalis*	Kynurenine, serotonergic, microbial system	*Lamiaceae*		BNDF, 5-HT, oxidative markers, and gut microbiota	Protects against neurotoxicity, improves depression, memory, behavioral activities, and the gut microbiota	[202,203,204]
	*Tagetes lucida*	Serotonergic system	*Asteraceae*		5-HT	Antidepressant, modulates 5-HT reuptake/release	[205,206,207]
	Tualang honey	Microbial, BDNF				Memory restoration and improvement in depression and cognitive and neural stresses	[208,209,210]
